# Hyperbaric Oxygen Therapy Represses the Warburg Effect and Epithelial–Mesenchymal Transition in Hypoxic NSCLC Cells *via* the HIF-1α/PFKP Axis

**DOI:** 10.3389/fonc.2021.691762

**Published:** 2021-07-21

**Authors:** Linling Zhang, Jingjing Ke, Shengping Min, Nan Wu, Fei Liu, Zhen Qu, Wei Li, Hongtao Wang, Zhongqing Qian, Xiaojing Wang

**Affiliations:** ^1^ Anhui Clinical and Preclinical Key Laboratory of Respiratory Disease, Molecular Diagnosis Center, Department of Pulmonary and Critical Care Medicine, First Affiliated Hospital of Bengbu Medical College, Bengbu, China; ^2^ Anhui Province Key Laboratory of Immunology in Chronic Diseases, Anhui Key Laboratory of Infection and Immunity, Department of Laboratory Medicine, Bengbu Medical College, Bengbu, China

**Keywords:** lung cancer, NSCLC, hyperbaric oxygen, HBO, Warburg effect, HIF-1

## Abstract

**Background:**

Tumor cells initiate hypoxia-induced mechanisms to fuel cell proliferation, invasion, and metastasis, largely mediated by low O_2_-responsive Hypoxia-Inducible Factor 1 Alpha (HIF-1α). Therefore, hyperbaric oxygen therapy (HBO) is now being studied in cancer patients, but its impact upon non-small-cell lung cancer (NSCLC) cell metabolism remains uncharacterized.

**Methods:**

We employed the NSCLC cell lines A549 and H1299 for *in vitro* studies. Glucose uptake, pyruvate, lactate, and adenosine triphosphate (ATP) assays were used to assess aerobic glycolysis (Warburg effect). A quantitative glycolytic flux model was used to analyze the flux contributions of HIF-1α-induced glucose metabolism genes. We used a Lewis lung carcinoma (LLC) murine model to measure lung tumorigenesis in C57BL/6J mice.

**Results:**

HBO suppressed hypoxia-induced HIF-1α expression and downstream HIF-1α signaling in NSCLC cells. One HIF-1α-induced glucose metabolism gene—Phosphofructokinase, Platelet (PFKP)—most profoundly enhanced glycolytic flux under both low- and high-glucose conditions. HBO suppressed hypoxia-induced PFKP transactivation and gene expression *via* HIF-1α downregulation. HBO’s suppression of the Warburg effect, suppression of hyperproliferation, and suppression of epithelial-to-mesenchymal transition (EMT) in hypoxic NSCLC cell lines is mediated by the HIF-1α/PFKP axis. *In vivo*, HBO therapy inhibited murine LLC lung tumor growth in a Pfkp-dependent manner.

**Conclusions:**

HBO’s repression of the Warburg effect, repression of hyperproliferation, and repression of EMT in hypoxic NSCLC cells is dependent upon HIF-1α downregulation. HIF-1α’s target gene PFKP functions as a central mediator of HBO’s effects in hypoxic NSCLC cells and may represent a metabolic vulnerability in NSCLC tumors.

## Introduction

Lung cancer is a leading cause of death worldwide (1). Non-small-cell lung cancer (NSCLC) accounts for approximately 85% of all lung-cancer related deaths and is associated with a poor patient prognosis (1). The poor prognosis is often due to late diagnosis, tumor metastasis, and resistance to conventional therapeutic strategies (2). Consequently, a better understanding of the underlying mechanism(s) responsible for NSCLC tumorigenesis and metastasis is needed to develop more effective diagnostic and therapeutic approaches.

Rapid proliferation of malignant cells coupled with abnormal angiogenesis creates low O_2_ partial pressures (hypoxia) within the local tumor microenvironment (3). As local O_2_ becomes scarce, tumor cells initiate hypoxia-induced mechanisms to fuel cell proliferation, invasion, and metastasis and well as promote angio- and vasculogenesis (3). These mechanisms are largely mediated by low O_2_-responsive Hypoxia-Inducible Factor 1 Alpha (HIF-1α) (4). During the hypoxic response, activated HIF-1α binds to hypoxia response elements (HREs) within the promoter region of target genes to promote aerobic glycolysis (the Warburg effect) and epithelial-to-mesenchymal transition (EMT) (5). These changes result in multiple cellular phenotypes that enhance malignant cell proliferation and aggressiveness (5).

Consequently, therapeutic strategies that can reduce tumor hypoxia such as hyperbaric oxygen therapy (HBO, 100% O_2_ at 1.5–3.0 bar) and normobaric oxygen therapy (NBO, 100% O_2_ at ambient pressure) may show promise in cancer patients (6). Moen et al.’s thorough review of the literature revealed that HBO therapy is safe with a low risk of complications and does not have a proliferative effect on malignant tumors (7). Moreover, HBO therapy can inhibit tumor growth in some cancers (e.g., breast carcinoma), but there is limited evidence of HBO’s efficacy in other cancer types (e.g., cervical and bladder carcinomas) (7). Currently, HBO is being more rigorously investigated as an adjuvant therapy to enhance the efficacy of conventional radio- and photodynamic therapies against certain solid tumors (8). Although clinical evidence regarding HBO therapy in NSCLC patients remains limited (9), HBO has been shown to rescue CoCl_2_-induced hypoxic NSCLC cells from dedifferentiation and apoptosis resistance *in vitro* (10). However, the mechanism(s) underlying HBO’s effects in hypoxic NSCLC cells remain unclear.

Here, we demonstrate that HBO’s repression of the Warburg effect, repression of hyperproliferation, and repression of EMT in hypoxic NSCLC cells is dependent upon HIF-1α downregulation. Furthermore, we discovered that a HIF-1α target gene—the glycolytic enzyme Phosphofructokinase, Platelet (PFKP, PFK1)—functions as a central mediator of HBO’s effects in hypoxic NSCLC cells. This study identifies PFKP as a critical factor in hypoxia/HIF-1α-induced NSCLC tumorigenesis.

## Materials and Methods

Ethical approval for all animal protocols was obtained in advance from the Ethics Committee at Bengbu Medical College (Bengbu, China). Animal protocols were carried out in line with the NIH Guide for the Care and Use of Laboratory Animals and in accordance with all institutional and governmental guidelines. The **Supplementary Material** details the procedures for cell culture, plasmid constructs and transfections, *in vitro* normobaric hypoxia and HBO therapy, glucose uptake, lactate and pyruvate assays, adenosine triphosphate (ATP) measurements, the Venn analysis to identify HIF-1α-induced glucose metabolism genes in hypoxic NSCLC cells, the glycolytic flux model, RNA extraction and quantitative real-time PCR (qPCR), immunoblotting, luciferase reporter assays, 5-ethynyl-2′-deoxyuridine (EdU) cell proliferation assays, migration and invasion assays, floating cell detachment assays, the *in vivo* HBO murine model of NSCLC, lung tumor analysis, immunofluorescence (IF) staining, and immunohistochemistry (IHC) staining. Unpaired Student’s *t*-tests (for two-group comparisons) or one-way analysis of variance (ANOVA) with Bonferroni post-hoc testing (for multiple group comparisons) were used for statistical analysis. Statistical significance was established at *P*-values of less than 0.05.

## Results

### HBO Suppresses Hypoxia-Induced HIF-1α Upregulation in NSCLC Cell Lines

HIFs are known to regulate glycolytic gene expression in response to hypoxia (11, 12). To investigate HIF expression in response to hypoxia and HBO in the NSCLC cell lines A549 and H1299, we discovered that HIF-1α expression (but not HIF-1β) was upregulated in response to hypoxia, which was downregulated by the addition of HBO (**Figure S1A**). We also confirmed that pcDNA.HIF1A plasmid transfection, but not the empty pcDNA control plasmid (pcDNA.Ctrl), was able to rescue HIF-1α expression in HBO-treated A549 and H1299 NSCLC cells (**Figure S1A**). qPCR analysis of the HIF-1α target genes Cbp/P300 Interacting Transactivator with Glu/Asp-Rich Carboxy-Terminal Domain 2 (CITED2) and vascular endothelial growth factor A (VEGFA) (13) confirmed hypoxia-induced HIF-1α signaling upregulation, HIF-1α signaling downregulation by HBO, and HIF-1α signaling rescue by pcDNA.HIF1A plasmid transfection (**Figure S1B**). In sum, HBO suppresses hypoxia-induced HIF-1α expression and downstream HIF-1α signaling in NSCLC cells.

### HBO Suppresses the Hypoxia-Induced Warburg Effect in NSCLC Cell Lines *via* HIF-1α

Clear links between hypoxia and enhanced aerobic glycolysis (the Warburg effect) have been previously shown across several cancer cell lines (14–16). However, the precise mechanism(s) by which hypoxia impacts the Warburg effect in NSCLC cells, and the effects of HBO upon this, remain largely uncharacterized.

**Figure 1:**
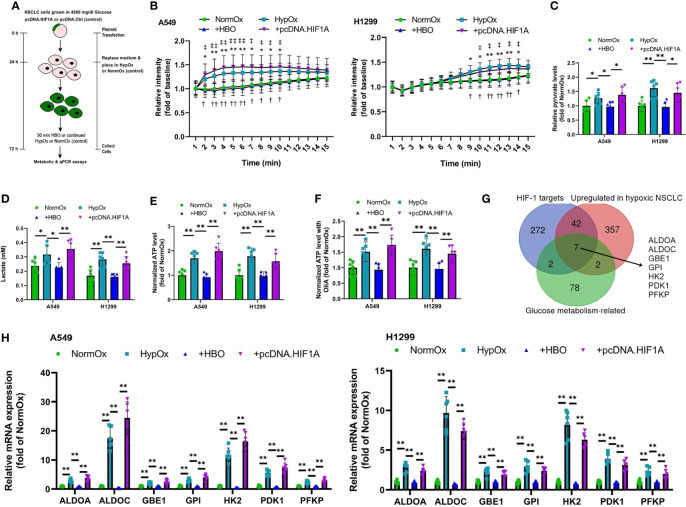
HBO suppresses the hypoxia-induced Warburg effect in NSCLC cell lines *via* HIF-1α. **(A)** A549 and H1299 NSCLC cells were subjected to hypoxia (HypOx) with or without hyperbaric oxygen (HBO) and pcDNA.HIF1A transfection. **(B)** Glucose uptake in A549 and H1299 cells. **(C)** Intracellular pyruvate levels in A549 and H1299 cells. **(D)** Lactate secretion in A549 and H1299 cells. **(E, F)** Intracellular ATP levels in A549 and H1299 cells incubated **(E)** without or **(F)** with oligomycin A for 1 h. **(G)** Venn analysis identifies seven HIF-induced, upregulated glucose metabolism genes in hypoxic NSCLC cells. **(H)** qPCR analysis of expression of the seven glucose metabolism genes identified in **(G)** in A549 and H1299 cells. Peptidylprolyl isomerase A (PPIA) was used as the housekeeping gene. Data represented as means ± SDs. n = 3 biological replicates × two technical replicates. *P <0.05, **P <0.01 [one-way ANOVA].

As elevated glucose uptake, enhanced production of the glycolytic metabolites lactate and pyruvate, and elevated ATP production are features of the Warburg effect (14–16), we measured glucose uptake, secretion of lactate into the supernatant, intracellular pyruvate levels, and intracellular ATP levels in A549 and H1299 NSCLC cells subjected to pcDNA.HIF1A or pcDNA.Ctrl plasmid transfection (**Figure 1A** and **Figure S1A**). HBO significantly reduced hypoxia-enhanced glucose uptake, supernatant levels of lactate, and intracellular pyruvate levels, effects rescued by HIF-1α overexpression (**Figures 1B–D**). NSCLC cells use aerobic glycolysis as their main source of ATP (17). In hypoxic cells, intracellular ATP levels were high (**Figure 1E**). Interestingly, intracellular ATP levels remained high following the addition of HBO, despite a reduction in glucose uptake (**Figure 1E**). This metabolic plasticity is common in cancer cells, as it allows them to switch between oxidative phosphorylation (OXPHOS) and glycolysis in response to their environment (18). To remove the cell’s ability to switch on OXPHOS, we pre-treated the NSCLC cells with oligomycin A, a specific inhibitor of mitochondrial ATP synthase (19). We found that, although intracellular ATP levels remained high in hypoxic cells, oligomycin A significantly reduced intracellular ATP levels following the addition of HBO (**Figure 1F**). This data suggests that hypoxic NSCLC cells are more dependent on OXPHOS to maintain intracellular ATP levels under HBO conditions.

The upregulation of key glucose metabolism genes is also a key feature of the Warburg effect (20). A recent RNA-sequencing study on A549 and HCC827 NSCLC cells comparing gene expression under either hypoxic or normoxic conditions revealed the activation of a shared hypoxia-induced gene program in these NSCLC cells (2). We performed a Venn analysis on this gene set to specifically identify HIF-1α-induced, upregulated genes in hypoxic NSCLC cells that participate in glucose metabolism. This Venn analysis identified seven glucose metabolism genes: Aldolase A (ALDOA), Aldolase C (ALDOC), Glycogen Branching Enzyme 1 (GBE1), Glucose Phosphate Isomerase (GPI), Hexokinase 2 (HK2), Pyruvate Dehydrogenase Kinase 1 (PDK1), and Phosphofructokinase, Platelet (PFKP, PFK1) (**Figure 1G**). Notably, five of these seven genes—ALDOA, ALDOC, GPI, PDK1, and PFKP—are significantly upregulated in lung adenocarcinoma (LUAD) patients from the Cancer Genome Atlas Lung Adenocarcinoma (TCGA-LUAD) cohort (**Figures S2A–E**). Moreover, elevated expressions of four of these seven genes—ALDOA, GBE1, GPI, and PFKP—are significantly associated with inferior survival in the TCGA-LUAD patient cohort (**Figures S3A–D**). We investigated the expression of these seven glycolytic markers in hypoxia/HBO-exposed A549 and H1299 NSCLC cells. HBO significantly reduced hypoxia-enhanced gene expression levels of all seven glycolytic markers, effects rescued by HIF-1α overexpression (**Figure 1H**). These findings reveal that HBO’s suppressive effects upon hypoxia-induced Warburg effect in NSCLC cells are mediated *via* HIF-1α downregulation.

### The HIF-1α Target Gene PFKP Controls Glycolytic Flux in NSCLC Cell Lines

**Figure 2:**
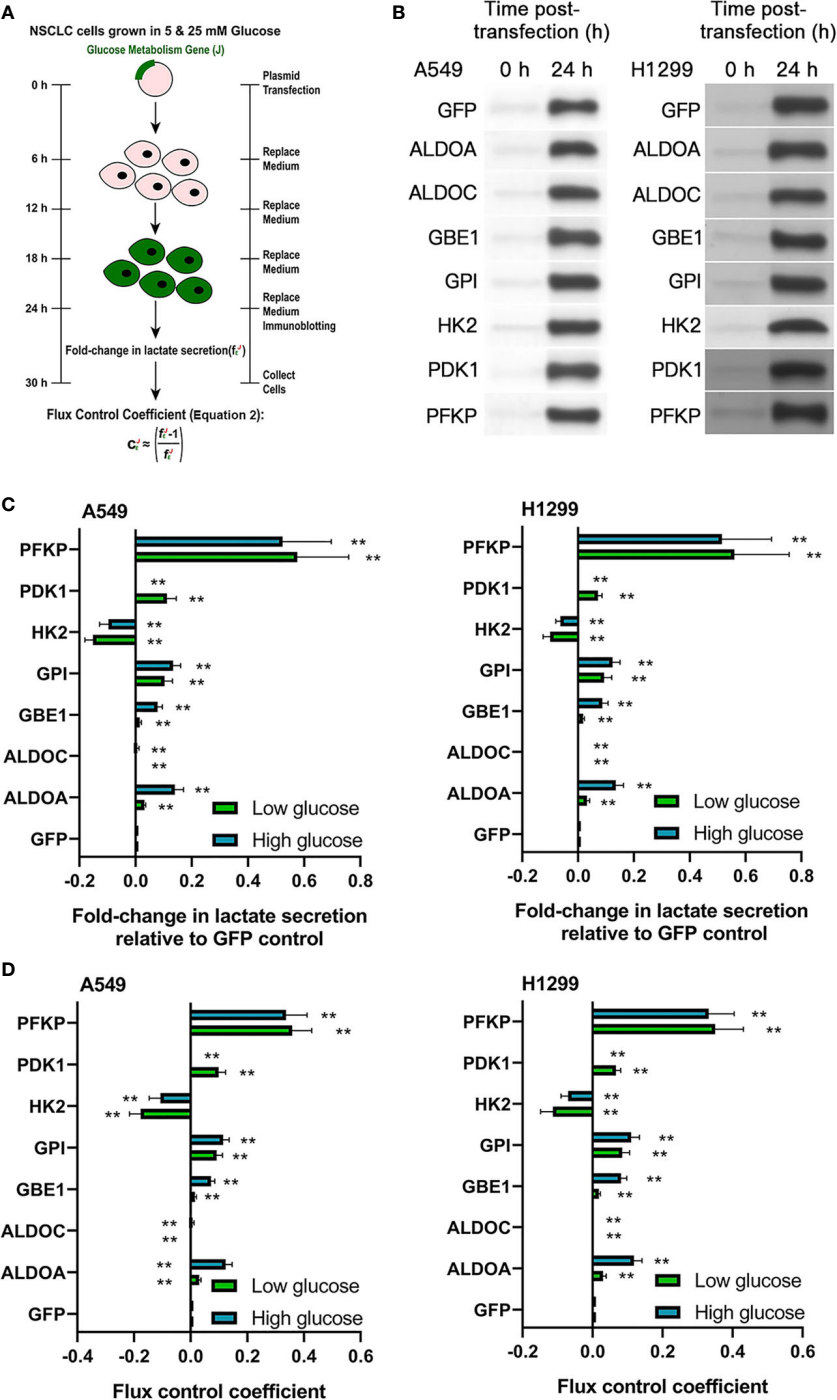
The HIF-1α target gene PFKP controls glycolytic flux in NSCLC cell lines. **(A)** Schematic of glycolytic flux experiments in A549 and H1299 NSCLC cells. The seven glucose metabolism genes identified in **Figure 1** were individually overexpressed in A549 and H1299 cells. Glycolytic flux was assessed from 24–30 h post-transfection with the f_E_ parameter: lactate secretion in gene-overexpressing cells/lactate secretion in GFP-expressing control cells. **(B)** Immunoblots validating gene overexpression in A549 and H1299 cells at 24 h post-transfection. **(C)** f_E_ values under low glucose (5 mM) conditions (green bars) or high glucose (25 mM) conditions (blue bars) and **(D)** associated flux-control coefficients (C_E_) defined in **(A)**. Data represented as means ± SDs. n = 3 biological replicates × two technical replicates. *P <0.05, **P <0.01 [unpaired Student’s *t*-test *vs*. glucose condition-matched GFP group].

We hypothesized that one or more of the seven HIF-induced glucose metabolism genes may be responsible for the aforementioned effects on Warburg metabolism in NSCLC cells. To identify which of the seven genes actually control glycolytic flux in NSCLC cells, we employed Tanner et al.’s glycolytic flux model (21) to independently overexpress each one of the seven HIF-induced glucose metabolism genes and green fluorescent protein (GFP) control in A549 and H1299 NSCLC cells (**Figure 2A**). We confirmed that GFP control transfection did not impact packed cell volume (PCV) or lactate secretion in A549 and H1299 cells (**Figures S4A, B**). Immunoblotting confirmed strong expression of each transfected gene construct at 24 h post-transfection (**Figure 2B** and **Figures S5A, B**). Glycolytic flux was measured from 24–30 h post-transfection under low (5 mM) and high (25 mM) glucose conditions. One of the seven overexpressed glucose metabolism genes—PFKP—most profoundly enhanced glycolytic flux under both low- and high-glucose conditions (**Figures 2C, D**). This evidence suggests that PFKP may underlie HBO’s suppressive effects upon hypoxia-induced Warburg phenotype in NSCLC cells and warrants further investigation.

### HBO Suppresses Hypoxia-Induced PFKP Upregulation in NSCLC Cell Lines *via* HIF-1α

**Figure 3:**
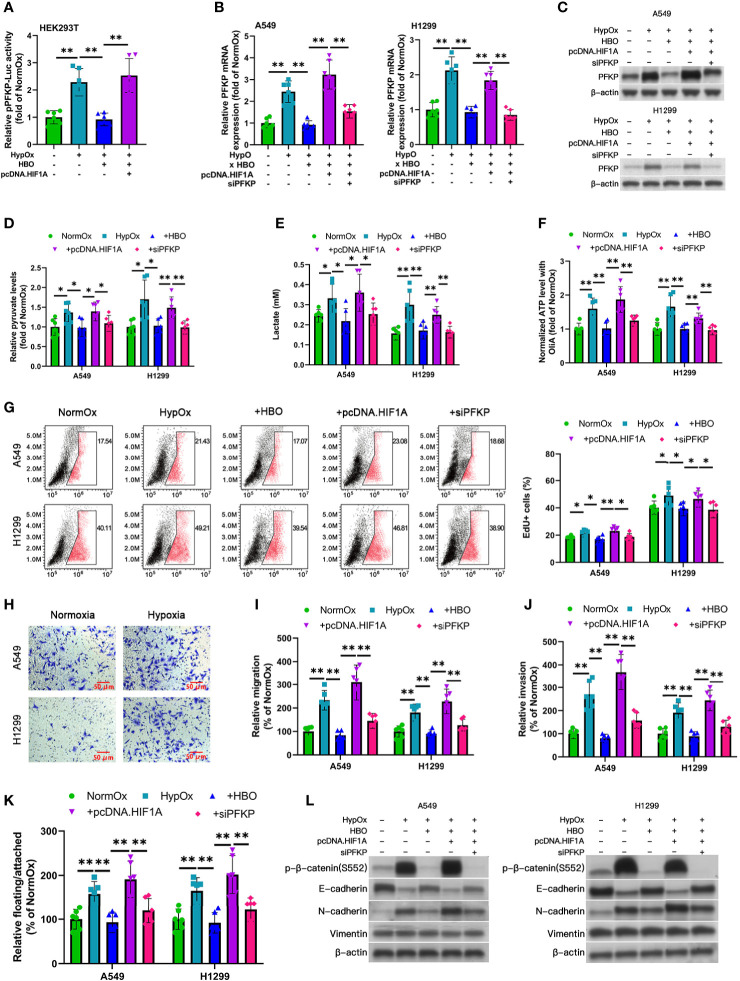
Reduced Warburg effect, hyperproliferation, and EMT in HBO-treated hypoxic NSCLC cell lines mediated by HIF-1α/PFKP axis. **(A)** PFKP-Luc reporter activity in HEK293T cells subjected to hypoxia (HypOx) with or without hyperbaric oxygen (HBO) and pcDNA.HIF1A. **(B–L)** A549 and H1299 NSCLC cells were subjected to hypoxia (HypOx) with or without hyperbaric oxygen (HBO) in the presence of pcDNA.HIF1A and siPFKP transfection. **(B, C)** Gene expression and protein levels of PFKP in A549 and H1299 cells. Peptidylprolyl isomerase A (PPIA) was used as the qPCR housekeeping gene. **(D)** Intracellular pyruvate levels in A549 and H1299 cells. **(E)** Lactate secretion in A549 and H1299 cells. **(F)** ATP levels in A549 and H1299 cells incubated with oligomycin A for 1 h. **(G)** EdU staining in A549 and H1299 cells. **(H)** Representative images of Transwell assays of cell migration. Scale bar = 50 μm. **(I, J)** Quantification of migration and invasiveness by Transwell assays in A549 and H1299 cells. **(K)** Quantification of detachment in A549 and H1299 cells. **(L)** Immunoblotting of p-β-catenin (S552), E-cadherin, N-cadherin, and vimentin in A549 and H1299 cells. Data represented as means ± SDs. n = three biological replicates × two technical replicates. *P <0.05, **P <0.01 [one-way ANOVA].

As HIF-1α directly binds to the PFKP promoter and transactivates PFKP (22), we investigated the role of HIF-1α in hypoxia and HBO’s effects upon PFKP transactivation and PFKP mRNA expression. HBO significantly reduced hypoxia-enhanced PFKP transactivation in HEK293T cells, which was rescued by HIF-1α overexpression (**Figure 3A**). HBO significantly reduced hypoxia-enhanced PFKP mRNA and protein expression in NSCLC cells, which was rescued by HIF-1α overexpression (**Figures 3B, C** and **Figure S6A**). Furthermore, the PFKP upregulation produced by rescue HIF-1α overexpression was abrogated upon siPFKP-mediated PFKP silencing (**Figures 3B, C** and **Figure S6A**). These findings reveal HBO’s suppressive effects upon hypoxia-induced PFKP transactivation and gene expression in NSCLC cells are mediated *via* HIF-1α downregulation.

### Reduced Warburg Effect, Hyperproliferation, and EMT in HBO-Treated Hypoxic NSCLC Cell Lines Mediated by HIF-1α/PFKP Axis

To determine the roles of HIF-1α and PFKP in HBO, we performed a series of gene modulation studies in hypoxia/HBO-exposed A549 and H1299 NSCLC cells. HIF-1α overexpression significantly enhanced lactate secretion and intracellular pyruvate levels, effects abrogated by the addition of siPFKP (**Figures 3D, E**). HIF-1α significantly enhanced intracellular ATP levels in oligomycin A-exposed cells, effects abrogated by the addition of siPFKP (**Figure 3F**).

As hypoxia promotes hyperproliferation and EMT in solid tumors (23), we investigated the impact of hypoxia and HBO on these factors in A549 and H1299 NSCLC cells. To examine NSCLC cell proliferation, we used EdU staining. HBO significantly reduced hypoxia-enhanced EdU + ve cell counts, effects rescued by HIF-1α overexpression (**Figure 3G**). This effect was abrogated by the addition of siPFKP (**Figure 3G**). HBO significantly reduced hypoxia-enhanced migration, invasiveness, and detachment, effects rescued by HIF-1α overexpression (**Figures 3H–K**). These effects were abrogated by the addition of siPFKP (**Figures 3H–K**). HBO significantly reduced hypoxia-enhanced the following EMT markers: p-β-catenin (S552) upregulation, E-cadherin downregulation, and N-cadherin upregulation; these effects were rescued by HIF-1α overexpression (**Figure 3L** and **Figures S6B, C**). These effects were abrogated by the addition of siPFKP (**Figure 3L** and **Figures S6B, C**). These findings reveal the reduced Warburg effect, hyperproliferation, and EMT in HBO-treated NSCLC cell lines following hypoxia is mediated by the HIF-1α/PFKP axis.

### HBO Therapy Inhibits Tumorigenesis *In Vivo* in a Pfkp-Dependent Manner

**Figure 4:**
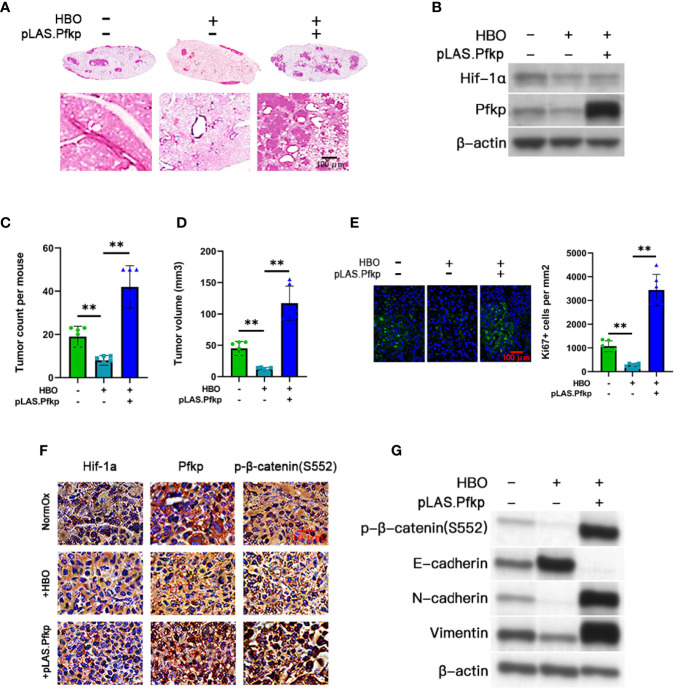
HBO therapy inhibits tumorigenesis *in vivo* in a Pfkp-dependent manner. Three cohorts were created in Lewis lung carcinoma (LLC) mice: (i) normoxic (NormOx) controls (n = 6), (ii) hyperbaric oxygen (HBO) therapy only (n = 6), and (iii) HBO therapy + pLAS.Pfkp (n = 6). **(A)** Representative images of hematoxylin and eosin (H&E)-stained whole lung sections (top) and higher-magnification lung sections (×400) (bottom). Scale bar = 100 μm. **(B)** Immunoblotting validation of Hif-1α and Pfkp. **(C)** LLC tumor counts and **(D)** mean LLC tumor volumes in each cohort. **(E)** Representative immunofluorescent images (×200) of Ki-67 (green)/Hoechst 33342 (blue) staining in LLC tumor sections and associated quantification of Ki-67+ cell counts per mm^2^ for each cohort. Scale bar = 100 μm. **(F)** Representative immunohistochemistry of Hif-1α, Pfkp, and p-β-catenin (S552) in LLC tumor sections (×100). Scale bar = 10 μm. **(G)** Immunoblotting of p-β-catenin (S552), E-cadherin, N-cadherin, and vimentin in LLC tumors. Data represented as means ± SDs. *P < 0.05, **P < 0.01 [one-way ANOVA].

To assess the role of PFKP in HBO therapy *in vivo*, we used a murine model of NSCLC to measure tumorigenesis in normoxic control or HBO-treated C57BL/6J mice tail-vein injected with control or Pfkp-overexpressing LLC cells (**Figure 4A**). We randomly assigned 18 mice into the three cohorts (n = 6 mice per cohort). Lungs containing LLC tumors were excised 14 days post-LLC cell injection. We confirmed that HBO therapy downregulated Hif-1α expression in LLC tumors; we also validated the presence of lentiviral-mediated Pfkp overexpression in pLAS.Pfkp LLC tumors (**Figure 4B** and **Figure S7A**). Lung tumor counts and mean tumor volumes were significantly reduced in HBO-treated mice, an effect abrogated by Pfkp overexpression (**Figures 4C, D**). IF staining confirmed a reduction in Ki-67 + ve cell counts in HBO-treated lung tumors, an effect abrogated by Pfkp overexpression (**Figure 4E**). IHC staining revealed Hif-1α, Pfkp, and p-β-catenin (S552) downregulation in HBO-treated lung tumors, effects abrogated by Pfkp overexpression (**Figure 4F**). Furthermore, immunoblotting revealed p-β-catenin (S552) downregulation, E-cadherin upregulation, N-cadherin downregulation, and vimentin downregulation in HBO-treated lung tumors, effects abrogated by Pfkp overexpression (**Figure 4G** and **Figure S7B**). Overall, these *in vivo* findings demonstrate that HBO therapy can inhibit murine lung tumor growth in a Pfkp-dependent manner.

## Discussion

Almost one century ago, Otto Warburg reported that cancer cells catabolize glucose into lactate under aerobic conditions (i.e., aerobic glycolysis or Warburg effect) (24). Modern cancer research has more precisely characterized this Warburg phenotype as a product of active metabolic reprogramming that supports cancer cell proliferation and progression within an unstable tumor microenvironment (20). Here, we demonstrate the existence of a HIF-1α/PFKP axis that supports the Warburg effect, hyperproliferation, and EMT in hypoxic NSCLC cells, an axis which is suppressed by HBO exposure. We also show that HBO therapy inhibits murine LLC lung tumor development in a Pfkp-dependent manner.

Warburg metabolic reprogramming is characterized by rapid ATP production to meet the energy demands of actively-proliferating cancer cells (20); specifically, the ATP production from the glucose-lactate conversion occurs 10–100 times faster than mitochondrial oxidation of glucose (25). Although NSCLC cells use OXPHOS as their main source of ATP (17), they maintain metabolic plasticity that allows them to produce ATP *via* glycolytic or OXPHOS programs depending on their environment (15, 26). Here, we showed that ATP levels remained high following the addition of HBO, despite a reduction in glucose uptake. However, when incubated with oligomycin A to block OXPHOS, ATP levels were significantly reduced following the addition of HBO. This evidence suggests that HBO promotes a shift from glycolysis to OXPHOS in hypoxic NSCLC cells to maintain high ATP levels under reduced glucose uptake conditions.

Warburg metabolic reprogramming is also characterized by upregulation of key glucose transporters and glycolytic enzymes to support enhanced glycolytic flux and glycolytic intermediate accumulation for biomass synthesis (20). Indeed, the Warburg effect has been attributed to the dysregulation of more than ten glycolytic enzymes (27). Here, we showed that HBO suppresses hypoxia/HIF-1α-induced upregulation of seven key glucose metabolism genes, one of which is involved in glycogen-glucose metabolism (GBE1) and six of which are directly involved in glycolysis (ALDOA, ALDOC, GPI, HK2, PDK1, and PFKP). We discovered that PFKP overexpression most profoundly enhanced glycolytic flux in NSCLC cells under both low- and high-glucose conditions (21). Associated with enhanced glycolytic activity, Warburg metabolic reprogramming is also characterized by lactate accumulation that promotes tumor progression (21). We showed that HBO suppresses hypoxia-induced upregulation of pyruvate and lactate *via* the HIF-1α/PFKP axis. Therefore, HBO appears to counteract Warburg metabolic reprogramming in NSCLC cells *via* the HIF-1α/PFKP axis.

The Warburg effect is directly linked to hyperproliferation in cancer cells. For instance, prominent glycolytic enzymes, such as LDHA and phosphoglycerate mutase 1 (PGAM1), have been shown to promote tumor cell proliferation (28). Moreover, metabolites produced in response to the Warburg effect, such as butyrate and acetyl-CoA, can affect histone acetylation of tumor suppressor genes and downstream cell proliferation (14, 29, 30). Here, we showed that HBO suppresses hypoxia-induced PFKP upregulation in NSCLC cells, and this PFKP downregulation functions as a critical mediator in HBO’s suppression of the Warburg effect as well as hyperproliferation and EMT. PFKP’s conversion of fructose-6-phosphate (F6P) to fructose-1,6-bisphosphate (F1,6BP) is the first rate-limiting step in mammalian glycolysis (31). Concordant with our findings, previous work also supports that PFKP promotes hyperproliferation and the Warburg phenotype in NSCLC cells cultured under normoxic conditions (32). Clinically, NSCLC and breast cancer tumors display PFKP upregulation, and high PFKP expression is associated with shorter patient survival in both cancers (32, 33). This combined evidence suggests that enhanced PFKP activity plays a central role in NSCLC tumorigenesis and HBO therapy may be an effective agent in suppressing aberrant PFKP activity in NSCLC tumors.

However, PFKP’s relationship with EMT is more complicated. PFKP has been shown to enhance EMT in breast cancer cells under hypoxic culture conditions (34). Lee et al. reports that EGFR-stimulated PFKP activity (via PI3K/AKT) enhances β-catenin (S552) phosphorylation and nuclear translocation that promotes EMT gene transcription (35, 36) in glioblastoma cells. Accordingly, we showed that PFKP overexpression promotes β-catenin (S552) phosphorylation and EMT marker expression in HBO-treated hypoxic NSCLC cells. However, under glucose-deprived conditions, the pro-EMT transcriptional regulator Snail has been shown to suppress PFKP to shunt glycolytic flux toward the pentose phosphate pathway to generate NADPH; PFKP downregulation under these conditions enhances cancer cell survival and metastasis (31). Since PFKP activity is subject to multiple regulatory signals (37), PFKP may play a pro-EMT role under normal glucose conditions but an anti-EMT role under glucose-deprived conditions. Further research across multiple cancer cell lines under various nutrient conditions is needed to thoroughly investigate this phenomenon.

As HBO therapy appears to be effective in suppressing aberrant PFKP activity in NSCLC cell lines as well as aberrant Pfkp activity in murine LLC lung tumors, our findings suggest that HBO therapy may be especially effective in NSCLC tumors (and perhaps other solid tumors) that display enhanced PFKP activity. In order to better pave the road for future clinical translation, further pre-clinical studies in this field should examine the effects of various HBO dosing regimens on lung tumor growth, HIF-1α and PFKP expression, EMT marker expression, metastatic spread, and survival rates in both orthotopic and autochthonous models of NSCLC.

In conclusion, we show that HBO’s repression of the Warburg effect, repression of hyperproliferation, and repression of EMT in hypoxic NSCLC cells is dependent upon HIF-1α downregulation. Furthermore, we discovered that the HIF-1α target gene PFKP functions as a central mediator of HBO’s effects in hypoxic NSCLC cells. This study identifies PFKP as a critical factor in hypoxia/HIF-1α-induced NSCLC tumor cell proliferation, invasion, and metastasis that may represent a metabolic vulnerability in NSCLC tumors. HIF-1α gene targets other than PFKP are known to regulate glycolysis, proliferation, and EMT Chen et al. (2). Furthermore, it is possible that HIF-1α-independent pathways may be involved in HBO’s effects on hypoxic NSCLC cells. Therefore, future studies should aim to identify other critical mediators of the Warburg effect, hyperproliferation, and EMT in hypoxic NSCLC cells.

## Data Availability Statement

The original contributions presented in the study are included in the article/**Supplementary Material**. Further inquiries can be directed to the corresponding author.

## Ethics Statement

The animal study was reviewed and approved in advance by the Ethics Committee at Bengbu Medical College (no. 2020-083; Bengbu, China).

## Author Contributions

Conceived and designed the study: XW, LZ, and JK. Performed the experimental procedures: LZ, JK, SM, NW, FL, and ZQ. Analyzed the data: WL, HW, and ZQQ. Drafted the manuscript: XW. All authors contributed to the article and approved the submitted version.

## Funding

This work was supported by the National Natural Science Foundation of China (82072585, 81772493), Project of Anhui Educational Committee for Distinguished Scholars (gxbjZD2020069), the 512 Talent Cultivation Project of Bengbu Medical College (by51201108), and the Anhui Provincial Major Science and Technology Project (202003a07020024).

## Conflict of Interest

The authors declare that the research was conducted in the absence of any commercial or financial relationships that could be construed as a potential conflict of interest.

## Abbreviations

EdU, 5-ethynyl-2′-deoxyuridine; ATP, adenosine triphosphate; ANOVA, analysis of variance; EMT, epithelial-to-mesenchymal transition; GFP, green fluorescent protein; HRP, horseradish peroxidase; HBO, hyperbaric oxygen therapy; HIF-1α, Hypoxia inducible factor 1-alpha; HREs, hypoxia response elements; IF, immunofluorescence; IHC, immunohistochemistry; LLC, Lewis lung carcinoma; NSCLC, Non-small-cell lung cancer; PCV, packed cell volume; PFKP, PFK1, Phosphofructokinase, Platelet; pPFKP-Luc, PFKP luciferase reporter.
