# Supplementary material for: Hyperbaric Oxygen Therapy Represses the Warburg Effect and Epithelial–Mesenchymal Transition in Hypoxic NSCLC Cells via the HIF-1α/PFKP Axis
Source: Front Oncol. 2021 Jul 21;11:691762. doi: 10.3389/fonc.2021.691762 (PMC8335162; doi:10.3389/fonc.2021.691762)
Supplement: Supplementary file 1 [file Presentation_1.pdf]

Supplementary Material

1 SUPPLEMENTARY METHODS

Cell culture

The human NSCLC lines A549 and H1299, the HEK293T cell line, and the murine Lewis lung carcinoma (LLC) cell line were purchased from American Type Culture Collection (ATCC). Cells were cultured in Dulbecco's Modified Eagle Medium (DMEM) supplemented with high glucose (4500 mg/l), L-glutamine (4 mM), fetal calf serum (10%), penicillin (100 U/ml), and streptomycin (100 mg/ml) unless otherwise specified. All cells were incubated in a humidified atmosphere (5% CO_2) at 37°C.

Plasmid constructs and transfections

For *in vitro* overexpression experiments**,** the sequence-verified human *HIF1A* cDNA (#MHS6278-202830081, Horizon Discovery) was inserted into a pcDNA4/HisMaxC mammalian expression plasmid (Thermo Fisher Scientific) via the *Eco*R1 and *Xho*1 sites. The resulting pcDNA.HIF1A construct, which was sequence-verified by Sanger sequencing (Genewiz), expresses the HIF-1α protein with an Xpress epitope tag at the N-terminus. Cells were plated in 12-well plates and transfected with either 400 ng empty pcDNA plasmid (pcDNA.Ctrl) or 400 ng pcDNA.HIF1A plasmid for 24 hours. For rescue siRNA transfections, cells were plated in 12-well plates and transfected with either Control siRNA-A (siCtrl, #sc-37007, SCBT) or *PFPK* siRNA (siPFPK, #sc-106401, SCBT) according to the manufacturer's instructions at day 3 post-pcDNA plasmid transfection.

For the *in vivo* murine model**,** the sequence-verified murine *Pfpk* cDNA (#MMM1013-202706114, Horizon Discovery) was inserted into the pLAS5w vector (RNAi Core of Academia Sinica, Taiwan) via the *Nhe*I and *Eco*R1 sites. Lentiviral particles were produced by transfecting HEK293T cells with empty pLAS vector or pLAS.Pfpk vector and packaging plasmids using TransIT-LT1 transfection reagent (Mirus Bio LLC). LLC cells were spin-infected by plating them in 12-well plates with the lentiviral supernatants and polybrene (8 µg/ml, Sigma-Aldrich), followed by 1100 g centrifugation for 30 min. LLC cells were subjected to selection with puromycin (3 ng/ml) to obtain stably-infected cells.

In vitro normobaric hypoxia and HBO therapy

Normobaric hypoxia was administered as previously described (Chen et al., 2019). Briefly, cells (1.0×10^4 cells/cm^2) were seeded under normal conditions and then incubated in a controlled 1% O_2 atmosphere in a hypoxia chamber (ASTEC, Japan) for 72 hours. For HBO exposure, hypoxic cells were positioned within a 27-l hyperbaric chamber (OXYCOM 250 ARC, HYPCOMOY, Finland). The hyperbaric chamber was flushed with pure O_2 gas at ambient pressure for 15 min. The pressure was gradually increased from 1.0 to 3.0 bar for 10 min and was then kept stable at 3.0 bar for 90 min. The chamber was flushed with pure O_2 gas for 5 min every 10 min. After the 90-min HBO period, the chamber was decompressed to 1.0 bar over 15 min.

Glucose uptake

Glucose uptake was measured in cells 72 hours post-transfection. Cells were plated in fresh complete media for 20 minutes before the glucose analog 2-NBDG (5 μM, Thermo Fisher Scientific) was added and left to incubate with the cells for an additional 15 minutes. After incubation, a microplate reader (excitation wavelength: 488 nm) was used to measure the fluorescence intensity of each sample.

Lactate secretion and intracellular pyruvate assays

The Glycolysis Cell Assay Kit (Cayman Chemicals) -- a colorimetric method for detecting L-lactate secreted by cultured cells --- was used to measure supernatant lactate levels as per the manufacturer's protocol.

For the intracellular pyruvate assays, cell lysates (5×10^5 cells) were incubated in pyruvate assay buffer (100 μl) from the Pyruvate Fluorometric Kit (BioVision) as per the manufacturer's protocol. Following incubation, lysates were centrifuged at 10000 g at 4°C for 10 minutes to remove insoluble debris. Supernatants were collected (50 μl) and loaded into a 96-well plate. Reaction mix was added (50 μl) and samples were left to incubated at room temperature for 30 minutes in the dark before a microplate reader (excitation/emission wavelengths: 535/590 nm) was used to measure the fluorescence intensity of each sample.

Intracellular adenosine triphosphate (ATP) measurements

Cells were isolated, then boiled in a 100°C water bath in pre-boiled ATP buffer (200 μl) for 2 minutes for the preparation of cell lysates. The lysates were then centrifuged for 2 minutes at 4°C before supernatants were collected and transferred to a new tube. BCA protein assays were used to determine protein levels, then the ATP Bioluminescence Assay Kit (Roche) was used as per the manufacturer's protocol. Intracellular ATP levels were detected using a microplate reader. Total protein levels were used to normalize ATP levels. For some experiments, cells were pre-treated with DMSO vehicle or oligomycin A (10 μg/ml in DMSO) for 60 min as previously described (Zhu et al., 2019).

Venn analysis to identify HIF-1α-induced glucose metabolism genes in hypoxic NSCLC cells

We performed a Venn analysis to specifically identify HIF-1α-induced, upregulated genes in hypoxic NSCLC cells that participate in glucose metabolism. HIF-1 gene targets were derived from Schödel et al.'s high-resolution genome-wide mapping of HIF-1 binding sites by chromatin immunoprecipitation linked to high throughput sequencing (ChIP-seq) (Schödel et al., 2011). Genes significantly upregulated by hypoxia in both A549 and HCC827 NSCLC cell lines were derived from Chen et al.'s *in vitro* studies (Chen et al., 2019). The glucose metabolism-related genes were derived from the Glucose Metabolism (SAB Target List) H96 gene panel (BioRad). A Venn analysis (http://bioinformatics.psb.ugent.be/webtools/Venn/) was performed to identify the union of all three gene sets.

Glycolytic flux model

We employed Tanner et al.'s glycolytic flux model with minor modifications (Tanner et al., 2018). Briefly, sequence-verified cDNAs of green fluorescent protein (GFP, #AG13105-UT, Sino Biological), Aldolase A (ALDOA, #MHS6278-202755903, Horizon Discovery), Aldolase C (ALDOC, #MHS6278-202759732, Horizon Discovery), Glycogen Branching Enzyme 1 (GBE1, #MHS6278-202831856, Horizon Discovery), Glucose Phosphate Isomerase (GPI, #MHS6278-202826268, Horizon Discovery), Hexokinase 2 (HK2, #MHS6278-202759113, Horizon Discovery), Pyruvate Dehydrogenase Kinase 1 (PDK1, #SC321678, Origene), and Phosphofructokinase, Platelet (PFKP, PFK1; #MHS6278-202800861, Horizon Discovery) were

independently cloned into pcDNA4/HisMaxC plasmids as described above. Cells were plated in 12-well plates and transfected with 400 ng of each pcDNA plasmid for 24 hours. To confirm gene overexpression, cells were subjected to immunoblotting at 24 hours post-transfection as described below.

Glucose and lactate measurements were conducted in six-well plates between 24 and 30 hours post-pcDNA plasmid transfection. At 24 hours post-transfection, cells were washed thrice with 1× PBS (2 ml) and then we added 1-ml volume of fresh DMEM containing 10% dialyzed FBS, glucose (5 mM or 25 mM), L-glutamine (0.584 g/l), phenol red (0.015 g/l), and sodium bicarbonate (3.7 g/l). At 30 hours post-transfection, a 300-ml supernatant volume was analyzed for glucose and lactate levels by a biochemical analyzer (HITACHI 7080, Japan) Changes in glucose and lactate were normalized to the calculated mean packed cell volume (PCV) during the six-hour assay period based on the final PCV value at 30 hours post-transfection and the calculated PCV growth rate (Figure S2A) using Tanner et al.'s Equation 3 (Tanner et al., 2018):

$$PCV_{Average} = \frac{\frac{1}{0.028h^{-1}}\left(PCV_{End} - \frac{PCV_{End}}{e^{0.028h^{-1} \times 6h}}\right)}{6h}$$

The flux control coefficient C^J_E for each overexpressed enzyme (*E)* was calculated using Tanner et al.'s Equation 2 (Tanner et al., 2018):

$$C^J_E \approx \left(\frac{f^J_E - 1}{f^J_E}\right)$$

where f^J_E is the simple fold-change in lactate secretion (f^J) produced by enzyme (*E)* overexpression (i.e., lactate secretion in enzyme *E*-overexpressing cells / lactate secretion in GFP-expressing control cells).

RNA extraction and qPCR

Trizol (Thermo Fisher Scientific) was used to isolated total RNA from cells as per the manufacturer's protocol. An EasyScript cDNA Synthesis Kit (TransGen, China) was used to generate cDNA from RNA. GoTaq qPCR Mix (Promega) was used to perform qPCR on the CFX96 Detection System (BioRad). All PCR primers were purchased from Origene; the primer sequences are available on the Origene website (www.origene.com). We used human

peptidylprolyl isomerase A (PPIA) as the housekeeping gene, as it has been shown to be stable under hypoxic conditions in two human cancer cell lines (Albuquerque et al., 2018). Gene expression data was generated using the $2^{-\Delta\Delta Ct}$ method.

Western blotting

Cell or tissue lysates were generated using RIPA buffer. BCA Protein Assays (BeyoTime Biotechnology, China) were used to quantify to amount of protein in each sample. Samples were then subjected to SDS-PAGE for separation before being heat transferred into PVDF membranes. Membranes were blocked at room temperature for 1 hour using skim milk (5%) in TBS-T. Next, membranes were washed and incubated at 4°C overnight with primary antibody, before being washed and incubated at room temperature for 1 hour the next day in secondary antibody. The primary antibodies were as follows: anti-HIF-1α (#sc-13515, SCBT), anti-HIF-1β (#MA1-515, Invitrogen), anti-GFP (#ab6556, Abcam), anti-ALDOA (#8060, CST), anti-ALDOC (#ab87122, Abcam), anti-GBE1 (#ab180596, Abcam), anti-GPI (#57893, CST), anti-HK2 (#2867, CST), anti-PDK1 (#5662, CST), anti-PFPK (#13389-1-AP, ProteinTech), anti-phospho-β-catenin(Ser552) (p-β-catenin(S552), #PA5-17154, Invitrogen), anti-E-cadherin (#3195, CST), anti-N-cadherin (ab18203, Abcam), anti-vimentin (ab92547, Abcam), and anti-β-actin (ab8227, Abcam). A gel imager (Tanon 5200, China) was used to quantify the proteins.

PFKP luciferase reporter assays

The PFKP luciferase reporter (pPFKP-Luc) was used for PFPK transactivation assays as previously reported (Liu et al., 2016). In brief, HEK293T cells were plated into 24 well plates, transfected with pPFKP-Luc and the internal control pTK-Renilla using VigoFect reagent (Vigorous Biotech, Beijing, China), and then cultured for 28 hours. Following hypoxia and/or HBO, luciferase assays were conducted in HEK293T cells using the Dual-Luciferase Reporter Assay System (Promega). Renilla luciferase activity was used to normalize pPFKP-Luc activity.

5-ethynyl-2′-deoxyuridine (EdU) cell proliferation assay

Cells were plated into six-well plates for 12 hours. EdU (20 µM) was added to the growth media and left to incubate for an additional three hours. Following incubation, cells were isolated and paraformaldehyde (4%) fixed for 20 minutes. Then, cells were permeabilized with Triton X-100 (0.5%) at room temperature for 10 minutes. The dye mix solution was added for a final 10 minute incubation at room temperature in the dark. Finally, the samples were ran on the flow cytometer (Accuri, BD Biosciences) to determine the percentage of EdU +ve cells.

Migration and invasion assays

Migration was assessed via Transwell assays (Corning Costar). Cells incubated in either normoxic or hypoxic conditions for 36 hours were subsequently serum-starved for a further 12 hours. Next, cells were placed in the top chamber in serum-free media, with 10% FBS media in the bottom chamber before being incubated for a further 24 hours. Cells on the upper membrane, which had not migrated, were collected. Cells on the bottom side of the membrane were paraformaldehyde (4%) fixed, stained in crystal violet (0.1%), and quantified via microscopy.

Cells were cultured in serum-free media in the top chamber of a Transwell assay covered with Matrigel. The bottom chamber was filled with 10% FBS media, and Transwells were incubated for 24 hours. Next, EdU was added and the cells were left for an additional 4 hours before membranes were removed and stained with the EdU kit (Invitrogen). Cell counting was then performed via microscopy.

Floating cell detachment assays

Floating cell detachment assays were performed as previously described (Cao et al., 2019). Briefly, cells were seeded into a 24-well plate and left to incubate for 12 hours at 37°C. Floating cells that did not attach were isolated by 250 g centrifugation for 5 min at room temperature, resuspended, stained with 0.4% trypan blue, and counted by hemocytometry with a bright-field microscope (200× magnification). Cells that had attached were counted following trypsinization. Detachment was calculated as the ratio of floating cells/attached cells.

***In vivo* HBO murine model of NSCLC**

Male C57BL/6J mice (6-7 weeks of age) were purchased from HuaFuKang Bioscience (China) and housed in plastic cages under a 12-h/12-h light/dark cycle with controlled temperature/humidity for one week prior to experimentation. Mice were fed commercial standard chow and water *ad libitum*. All efforts were made to minimize animal suffering, and all mice were euthanized by cervical dislocation.

Stably-infected LLC cells (5.0×10^5 cells in PBS) were tail vein-injected into male C57BL/6J mice (7-8 weeks of age) to establish the NSCLC model. HBO therapy was performed as previously described (Yttersian Sletta et al., 2017) with minor modifications. Briefly, mice were positioned within a 27-l hyperbaric chamber (OXYCOM 250 ARC, HYPCOMOY, Finland). The hyperbaric chamber was flushed with pure O_2 gas at ambient pressure for 15 min. The pressure was gradually increased from 1.0 to 2.5 bar for 10 min and was then kept stable at 2.5 bar for 90 min. The chamber was flushed with pure O_2 gas for 5 min every 10 min. After the 90-min HBO period, the chamber was decompressed to 1.0 bar over 15 min. The mice underwent this HBO procedure on days 1, 4, 7, 10 and 13 post-LLC cell injection. Controls were exposed to normoxia at 1.0 bar under otherwise identical conditions. Mice were monitored during the course of the experiment. At day 14 post-LLC cell injection, mice were humanely sacrificed. The lungs were excised for analysis.

Lung tumor analysis

The gross morphology of the excised lungs and the number of lung tumor nodules were assessed. Tumor sizes were measured with calipers; total tumor volumes were calculated as follows: $0.5236 \times (d_1 \times d_2 \times d_3)$, where the $d_{1\text{-}3}$ parameters represent the three orthogonal diameters. The lungs were inflated and 10% formalin-fixed at 25 cm H_2O pressure before pathohistological staining. Hematoxylin and eosin (H&E)-stained slides of fixed tumor tissues were scanned with an Aperio Scan Scope XT Slide Scanner.

Immunofluorescence (IF) staining

The fixed lung tumor samples were paraffin-embedded before being sliced into thin sections for IF staining. Sections were heated in citrate buffer (pH 6.0, 0.01 M) in a water bath (95°C) for 10 minutes, then treated with hydrogen peroxide (30%). Sections were then blocked with BSA (10% in PBA) for 60 minutes

before incubation with Alexa Fluor® 488-conjugated anti-Ki-67 (#11882, CST) overnight. Hoeschst was used to counterstain nuclei before sections were mounted using mounting media (Thermo Fisher Scientific). The Evos Auto Cell Imager (Thermo Fisher Scientific) was used to visualize Ki-67 +ve cells. ImagePro software was used for quantification.

Immunohistochemistry (IHC) staining

The fixed lung tumor samples were paraffin-embedded and sectioned as described above. Sections were blocked with BSA (10%), then stained overnight with the anti-HIF-1α, anti-PFPK, anti-p-β-catenin(S552) primary antibodies described above. Next, sections were stained at room temperature for 30 minutes with a secondary horseradish peroxidase (HRP)-conjugated antibody. Finally, DAB and hematoxylin were used as a counterstain. The Evos Auto Cell Imager (Thermo Fisher Scientific) was used to visualize staining.

Statistics

SPSS software was used to perform all statistical analyses. Unpaired Student's *t*-tests (for two-group comparisons) or one-way analysis of variance (ANOVA) with Bonferroni post-hoc testing (for multiple group comparisons) were used for statistical analysis. Statistical significance was established at *P*-values of less than 0.05 for all analyses.

2 SUPPLEMENTARY FIGURES

Figure S1. HBO rescues hypoxia-induced HIF-1α upregulation in NSCLC cell lines

A549 and H1299 NSCLC cells were subjected to hypoxia (HypOx) with or without hyperbaric oxygen (HBO) and pcDNA.HIF1A transfection. **(A)** HIF-1α and HIF-1β protein levels in A549 and H1299 cells. **(B)** qPCR analysis of HIF-1α target gene expression in A549 and H1299 cells. Data represented as means ± SDs. n=3 biological replicates × 2 technical replicates. **P*<0.05, ***P*<0.01 [one-way ANOVA].

Figure S2. Differential expression analysis of the seven glucose metabolism genes in the TCGA-LUAD patient cohort

Differential expression analysis of the seven glucose metabolism genes in TCGA-LUAD lung tumor samples (n=515) versus normal lung tissue samples (n=59). Box plots showing significantly higher mRNA expression of **(A)** ALDOA, **(B)** ALDOC, **(C)** GPI, **(D)** PDK1, and **(E)** PFKP in lung tumor samples versus normal lung tissue samples.

Figure S3. Survival analysis of the seven glucose metabolism genes in the TCGA-LUAD patient cohort

Survival analysis of the seven glucose metabolism genes in TCGA-LUAD lung tumor samples based on high gene expression (top quartile) versus low/medium gene expression (bottom three quartiles). Kaplan-Meier survival curves showing significantly inferior survival rates in patients with lung tumor samples displaying high gene expression of **(A)** ALDOA, **(B)** GBE1, **(C)** GPI, and **(D)** PFKP.

Figure S4. Transfection of GFP control vector does not influence cell proliferation or lactate secretion in NSCLC cell lines

A549 and H1299 NSCLC cells were transfected with the GFP-expressing control vector. **(A)** Cell proliferation measured by packed cell volume (PCV) in non-transfected control (NT Ctrl) cells,

empty pcDNA-transfected (pcDNA.Ctrl) cells, and GFP-expressing (pcDNA.GFP) cells over 36 hours post-transfection. **(B)** Lactate secretion in NT Ctrl, pcDNA.Ctrl, and pcDNA.GFP between 24 and 30 hours post-transfection normalized to PCV at 30 hours post-transfection. Data represented as means ± SDs. n=3 biological replicates × 2 technical replicates. **P*<0.05, ***P*<0.01 [one-way ANOVA].

Figure S5. Densitometric analyses associated with Figure 2B

The seven glucose metabolism genes identified in **Figure 1** were individually overexpressed in A549 and H1299 cells. Densitometric analysis of immunoblots validating gene overexpression in **(A)** A549 and **(B)** H1299 cells at 24 hours post-transfection. Data represented as means ± SDs. n=3 biological replicates × 2 technical replicates. **P*<0.05, ***P*<0.01 [unpaired Student's *t*-test].

Figure S6. Densitometric analyses associated with Figure 3C and Figure 3L

(A) Densitometric analysis of PFKP immunoblots depicted in **Figure 3C**. **(B, C)** Densitometric analysis of immunoblots depicted in **Figure 3L** for p-β-catenin(S552), E-cadherin, N-cadherin, and vimentin in **(B)** A549 and **(C)** H1299 cells. Data represented as means ± SDs. n=3 biological replicates × 2 technical replicates. **P*<0.05, ***P*<0.01 [one-way ANOVA].

Figure S7. Densitometric analyses associated with Figure 4B and Figure 4G

Three cohorts were created in Lewis lung carcinoma (LLC) mice: (i) normoxic (NormOx) controls (n=6), (ii) hyperbaric oxygen (HBO) therapy only (n=6), and (iii) HBO therapy + pLAS.Pfkp (n=6). **(A)** Densitometric analysis of immunoblots depicted in **Figure 4B** for Hif-1α and Pfkp. **(B)** Densitometric analysis of immunoblots depicted in **Figure 4G** for p-β-catenin(S552), E-cadherin, N-cadherin, and vimentin in LLC tumors. Data represented as means ± SDs. **P*<0.05, ***P*<0.01 [one-way ANOVA].

Figure S1

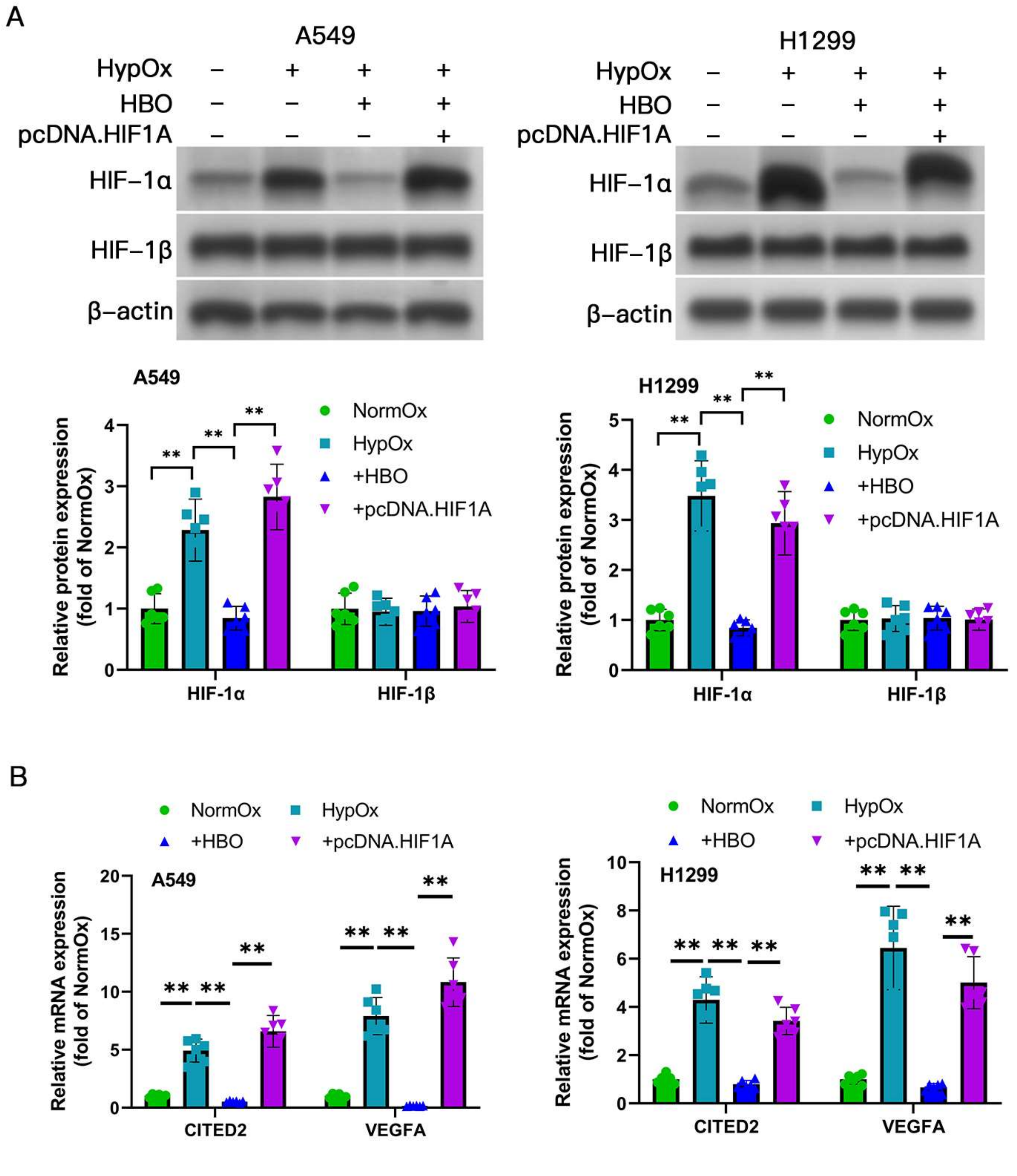
A
A549
HypOx
HBO
pcDNA.HIF1A
HIF-1α
HIF-1β
β-actin
H1299
HypOx
HBO
pcDNA.HIF1A
HIF-1α
HIF-1β
β-actin
A549
NormOx
HypOx
+HBO
+pcDNA.HIF1A
Relative protein expression (fold of NormOx)
HIF-1α
HIF-1β
H1299
NormOx
HypOx
+HBO
+pcDNA.HIF1A
Relative protein expression (fold of NormOx)
HIF-1α
HIF-1β
B
NormOx
HypOx
+HBO
+pcDNA.HIF1A
A549
Relative mRNA expression (fold of NormOx)
CITED2
VEGFA
NormOx
HypOx
+HBO
+pcDNA.HIF1A
H1299
Relative mRNA expression (fold of NormOx)
CITED2
VEGFA

Figure S2

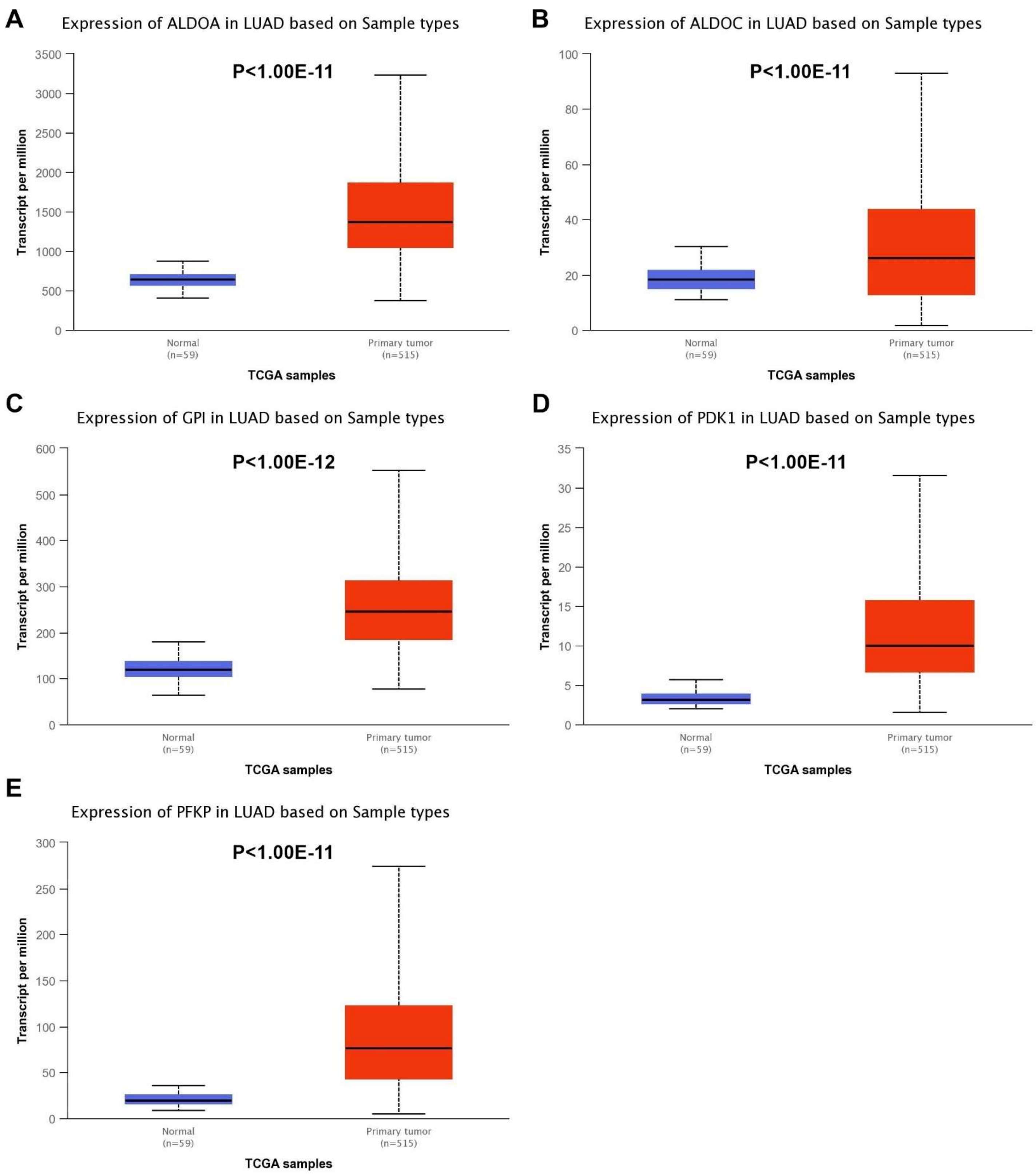
A
Expression of ALDOA in LUAD based on Sample types
P<1.00E-11
Transcript per million
0
500
1000
1500
2000
2500
3000
3500
Normal
(n=59)
Primary tumor
(n=515)
TCGA samples
B
Expression of ALDOC in LUAD based on Sample types
P<1.00E-11
Transcript per million
0
20
40
60
80
100
Normal
(n=59)
Primary tumor
(n=515)
TCGA samples
C
Expression of GPI in LUAD based on Sample types
P<1.00E-12
Transcript per million
0
100
200
300
400
500
600
Normal
(n=59)
Primary tumor
(n=515)
TCGA samples
D
Expression of PDK1 in LUAD based on Sample types
P<1.00E-11
Transcript per million
0
5
10
15
20
25
30
35
Normal
(n=59)
Primary tumor
(n=515)
TCGA samples
E
Expression of PFKP in LUAD based on Sample types
P<1.00E-11
Transcript per million
0
50
100
150
200
250
300
Normal
(n=59)
Primary tumor
(n=515)
TCGA samples

Figure S3

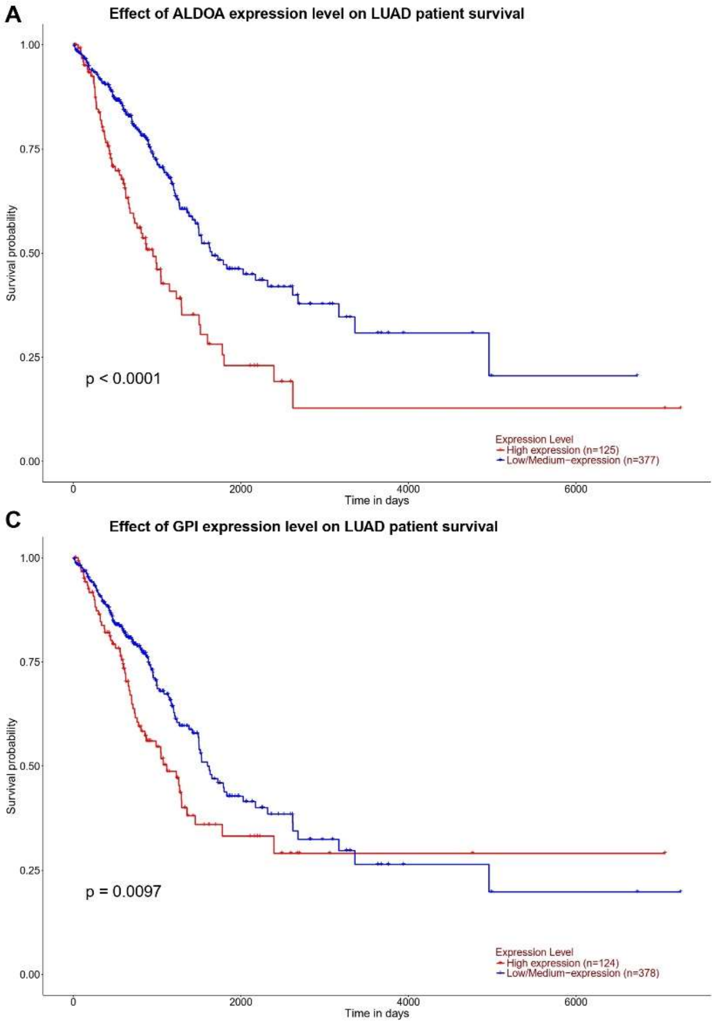
A
Effect of ALDOA expression level on LUAD patient survival
p < 0.0001
Expression Level
High expression (n=125)
Low/Medium−expression (n=377)
B
Effect of GBE1 expression level on LUAD patient survival
p < 0.0001
Expression Level
High expression (n=125)
Low/Medium−expression (n=377)
C
Effect of GPI expression level on LUAD patient survival
p = 0.0097
Expression Level
High expression (n=124)
Low/Medium−expression (n=378)
D
Effect of PFKP expression level on LUAD patient survival
p = 0.00061
Expression Level
High expression (n=125)
Low/Medium−expression (n=377)
Survival probability
Time in days
1.00
0.75
0.50
0.25
0.00
0
2000
4000
6000

Figure S4

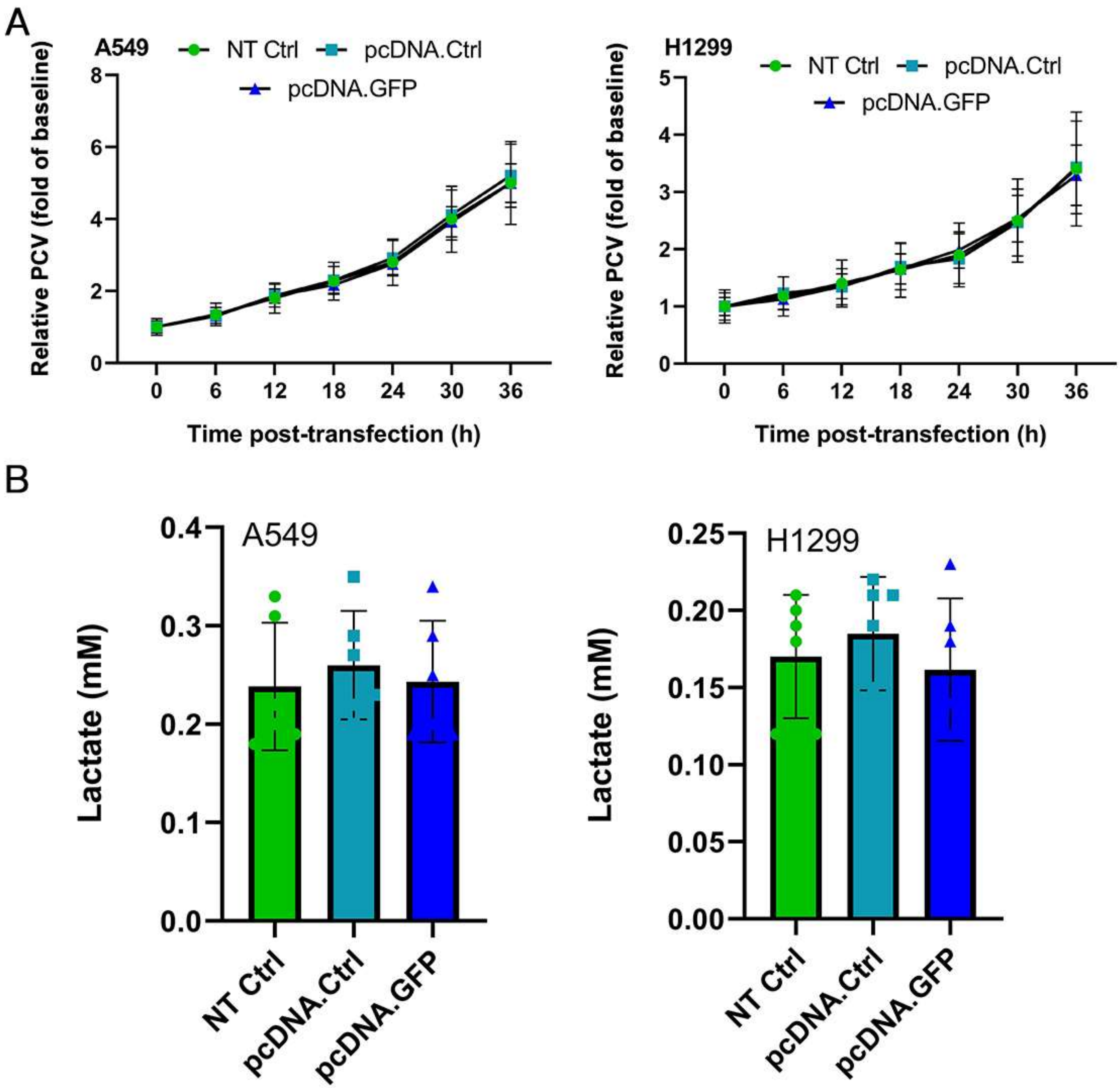
A
A549
NT Ctrl
pcDNA.Ctrl
pcDNA.GFP
Relative PCV (fold of baseline)
Time post-transfection (h)
H1299
B
Lactate (mM)
A549
H1299
NT Ctrl
pcDNA.Ctrl
pcDNA.GFP

Figure S5

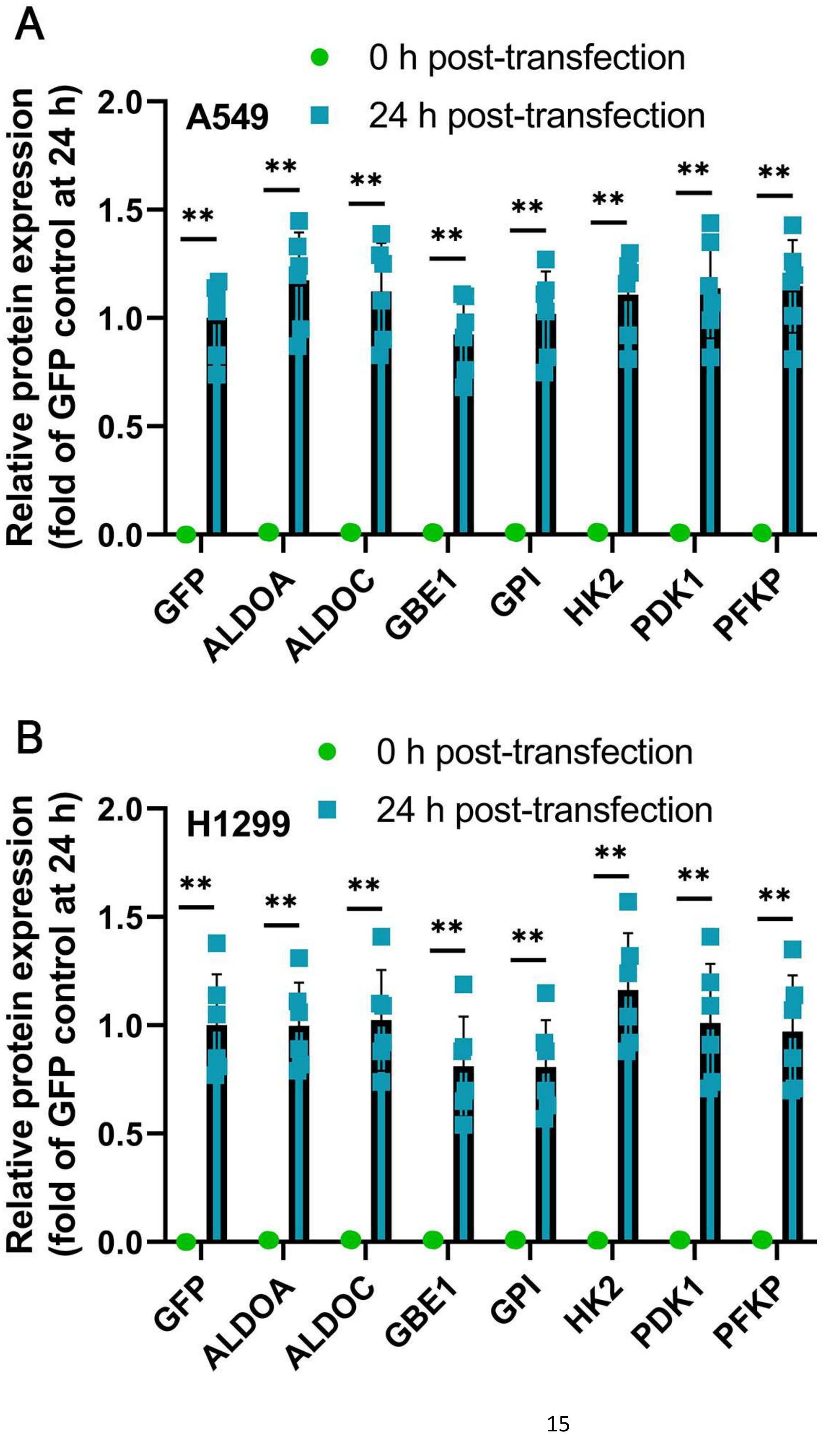
A
0 h post-transfection
24 h post-transfection
A549
Relative protein expression (fold of GFP control at 24 h)
2.0
1.5
1.0
0.5
0.0
**
GFP
ALDOA
ALDOC
GBE1
GPI
HK2
PDK1
PFKP
B
0 h post-transfection
24 h post-transfection
H1299
Relative protein expression (fold of GFP control at 24 h)
2.0
1.5
1.0
0.5
0.0
**
GFP
ALDOA
ALDOC
GBE1
GPI
HK2
PDK1
PFKP

Figure S6

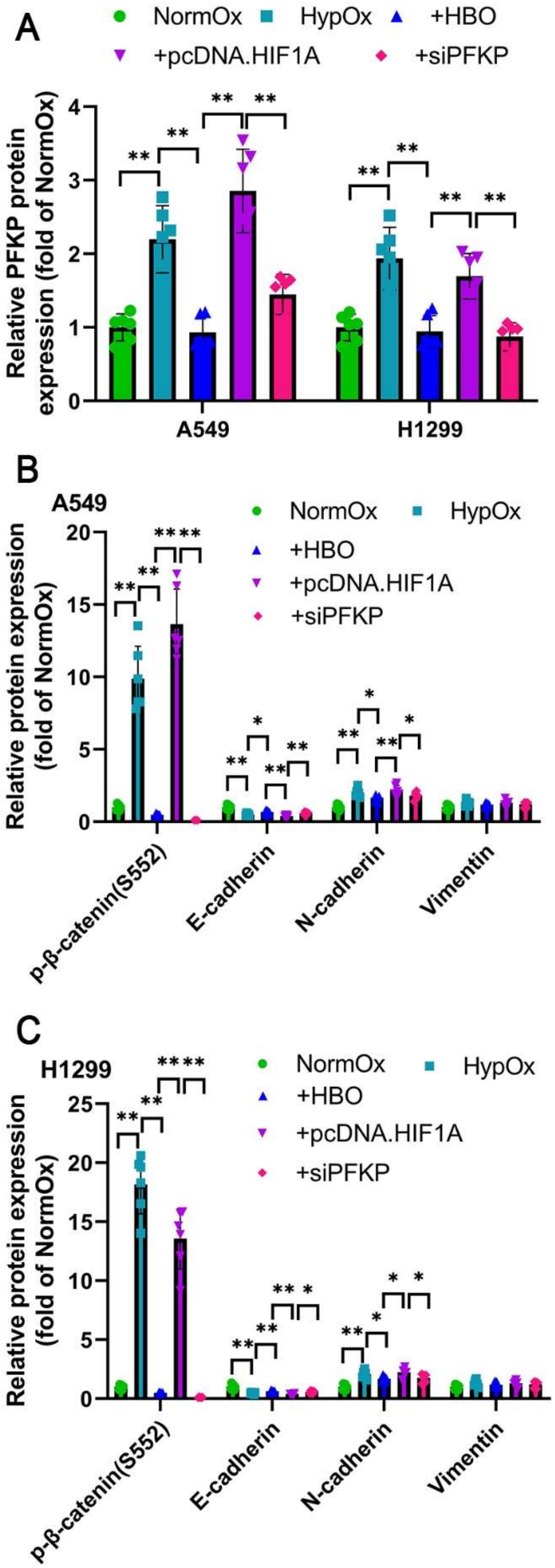
A
NormOx
HypOx
+HBO
+pcDNA.HIF1A
+siPFKP
Relative PFKP protein expression (fold of NormOx)
A549
H1299
B
A549
Relative protein expression (fold of NormOx)
p-β-catenin(S552)
E-cadherin
N-cadherin
Vimentin
C
H1299

Figure S7

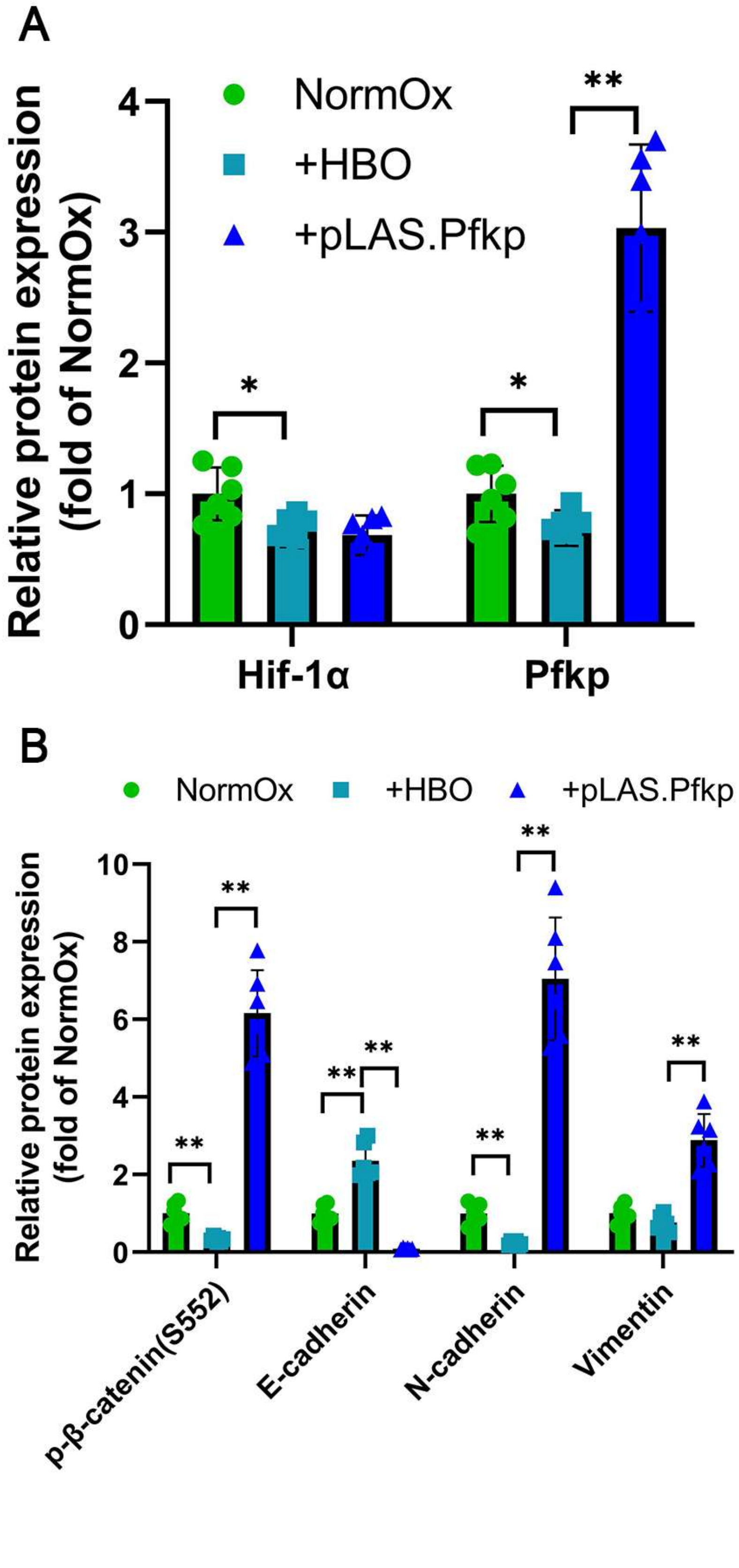

A
NormOx
+HBO
+pLAS.Pfkp
Relative protein expression (fold of NormOx)
4
3
2
1
0
**
*
*
Hif-1α
Pfkp
B
NormOx
+HBO
+pLAS.Pfkp
Relative protein expression (fold of NormOx)
10
8
6
4
2
0
**
p-β-catenin(S552)
E-cadherin
N-cadherin
Vimentin

REFERENCES FOR SUPPLEMENTARY MATERIAL

Albuquerque, A.P., Balmana, M., Reis, C.A., and Beltrao, E.I. (2018). Identification of appropriate housekeeping genes for quantitative RT-PCR analysis in MDA-MB-231 and NCI-H460 human cancer cell lines under hypoxia and serum deprivation. *Journal of Molecular and Clinical Medicine* 1**,** 127-134.

Cao, Y., Wang, X., Li, Y., Evers, M., Zhang, H., and Chen, X. (2019). Extracellular and macropinocytosis internalized ATP work together to induce epithelial–mesenchymal transition and other early metastatic activities in lung cancer. *Cancer Cell International* 19**,** 254.

Chen, Y.L., Zhang, Y., Wang, J., Chen, N., Fang, W., Zhong, J., Liu, Y., Qin, R., Yu, X., and Sun, Z. (2019). A 17 gene panel for non‐small‐cell lung cancer prognosis identified through integrative epigenomic‐transcriptomic analyses of hypoxia‐induced epithelial–mesenchymal transition. *Molecular oncology* 13**,** 1490-1502.

Liu, X., Cai, X., Hu, B., Mei, Z., Zhang, D., Ouyang, G., Wang, J., Zhang, W., and Xiao, W. (2016). Forkhead transcription factor 3a (FOXO3a) modulates hypoxia signaling via up-regulation of the von Hippel-Lindau gene (VHL). *Journal of Biological Chemistry* 291**,** 25692-25705.

Schödel, J., Oikonomopoulos, S., Ragoussis, J., Pugh, C.W., Ratcliffe, P.J., and Mole, D.R. (2011). High-resolution genome-wide mapping of HIF-binding sites by ChIP-seq. *Blood, The Journal of the American Society of Hematology* 117**,** e207-e217.

Tanner, L.B., Goglia, A.G., Wei, M.H., Sehgal, T., Parsons, L.R., Park, J.O., White, E., Toettcher, J.E., and Rabinowitz, J.D. (2018). Four key steps control glycolytic flux in mammalian cells. *Cell systems* 7**,** 49-62. e48.

Yttersian Sletta, K., Tveitarås, M.K., Lu, N., Engelsen, A.S., Reed, R.K., Garmann-Johnsen, A., and Stuhr, L. (2017). Oxygen-dependent regulation of tumor growth and metastasis in human breast cancer xenografts. *PloS one* 12**,** e0183254.

Zhu, B., Cao, X., Zhang, W., Pan, G., Yi, Q., Zhong, W., and Yan, D. (2019). MicroRNA‐31‐5p enhances the Warburg effect via targeting FIH. *The FASEB Journal* 33**,** 545-556.
